# Meta-Analysis of Randomized Controlled Trials on the Efficacy of Di'ao Xinxuekang Capsule and Isosorbide Dinitrate in Treating Angina Pectoris

**DOI:** 10.1155/2012/904147

**Published:** 2012-02-05

**Authors:** Yongliang Jia, Cong Chen, Choi-San Ng, Siu-Wai Leung

**Affiliations:** ^1^State Key Laboratory of Quality Research in Chinese Medicine, University of Macau, Taipa 999078, Macau; ^2^Institute of Chinese Medical Sciences, University of Macau, Taipa 999078, Macau; ^3^BIGHT Laboratory, School of Informatics, University of Edinburgh, Edinburgh EH8 9AB, UK

## Abstract

*Objective*. Randomized controlled trials (RCTs) on di'ao xinxuekang capsule (XXK) in treating angina pectoris were published only in Chinese and have not been systematically reviewed particularly for comparing XXK with isosorbide dinitrate (ISDN). This study aims to provide a comprehensive PRISMA compliant and internationally accessible systematic review and meta-analysis to evaluate the efficacies of XXK and ISDN in treating angina pectoris. *Methods*. The RCTs published between 1989 and 2011 on XXK and ISDN in treating angina pectoris were selected according to specific criteria. Meta-analysis was performed to evaluate the symptomatic (SYMPTOMS) and electrocardiographic (ECG) improvements after treatment. Odds ratios (OR) were used to measure effect sizes. Subgroup analysis, sensitivity analysis, and metaregression were conducted to evaluate the robustness of the results. *Results*. Seven RCTs with 550 participants were eligible. Overall ORs for comparing XXK with ISDN were 4.11 (95% CI :  2.57, 6.55) in SYMPTOMS and 2.37 (95% CI : 1.46, 3.84) in ECG. Subgroup analysis, sensitivity analysis, and metaregression found no significant dependence of overall ORs upon specific study characteristics. *Conclusion*. The meta-analysis of eligible but limited RCTs demonstrates that XXK seems to be more effective than ISDN in treating angina pectoris. Further RCTs of high quality are warranted to be conducted for update of the results of this meta-analysis.

## 1. Introduction

Ischemic heart disease, an imbalance coronary blood flow and myocardial requirement [1], usually has symptomatic discomfort in the chest, that is, angina pectoris [2], due to coronary insufficiency and myocardium ischemia and hypoxia [3]. Nitrates, calcium channel blockers, beta-receptor blockers are common drugs for treating angina pectoris [4]. Isosorbide dinitrate (ISDN) is one of the most effective and frequently used nitrates for treating angina pectoris [3, 5]. Di'ao xinxuekang (XXK) capsule is a second-class new drug of China for treating angina pectoris [6]. The main components of XXK are extracted from the rhizomes of *Dioscorea panthaica Prain et Burkill* and *Dioscorea nipponica Makino* [7].

 Randomized controlled trials (RCTs) on treatments of angina pectoris found that XXK was more effective than aspirin [8] and more effective than some Chinese medicinal formulations including compound danshen, guanxin suquan pills, and danshen dripping pills [9, 10]. A previous systematic review published in 2010 [11] suggested that XXK were more effective than danshen tablet but did not cite any eligible RCT and did not report any subgroup or sensitivity analysis. XXK and danshen dripping pills are two popular Chinese medicinal products often compared with ISDN in treating angina pectoris [12–15]. A recent systematic review published in 2011 [16] indicated that danshen dripping pills was more effective than ISDN. Furthermore, there is no systematic review or meta-analysis of RCTs comparing XXK with conventional nitrates such as ISDN. As such, it is not clear about whether XXK is more effective than ISDN in treating angina pectoris. This study aims to conduct a comprehensive, PRISMA-compliant [17], internationally accessible, and timely meta-analysis, with subgroup and sensitivity analyses of RCTs to compare the efficacies of XXK and ISDN in treating angina pectoris.

## 2. Methods

### 2.1. Study Selection Criteria

RCTs published in Chinese or English between 1989 (i.e., the year of XXK launch) and 2011 on comparing XXK with ISDN in treating angina pectoris were screened. Inclusion criteria were as follow: (a) participants were suffering from and being treated for angina pectoris; (b) the study was claimed to be an RCT; (c) the study compared XXK with ISDN in efficacy; (d) duration of treatment (follow-up period) was at least four weeks.

 Exclusion criteria were as follow: (a) duplicated or redundant study; (b) XXK was used in combination with other drugs; (c) ISDN was used in combination with other drugs; (d) the study did not include symptomatic (SYMPTOMS) improvement as the major outcome.

### 2.2. Search Strategies

RCT reports published between the 1989 and 2011 were searched from databases including China National Knowledge Infrastructure (CNKI), WanFang Data, China Master Theses Full-text Database (CMTD), China Doctor Dissertations Full-text Database (CDMD), Medline, PubMed, Cochrane Library, ScienceDirect and EMbase. The last search was performed on 22 August 2011.

 The search strategy was formulated mainly for searching xinxuekang because of few publications on the drug. For instance, WanFang Data was searched with such a simple query as: ((“guanxinbing (heart disease)” in title or “xinjiaotong (angina pectoris)” in title) and “xinxuekang” in abstract). The exact search strategies and query syntax were slightly adjusted according to the users' instructions from different databases.

### 2.3. Study Selection

Two reviewers (Y. Jia and C. Chen) independently screened all retrieved studies according to the eligibility criteria. Disagreement between reviewers was resolved by consensus. A flow diagram of study selection was generated according to the PRISMA requirements [17].

### 2.4. Data Extraction

Two reviewers (Y. Jia and C. Ng) independently extracted data items comprised (a) publication year; (b) number of authors; (c) trial dates; (d) baseline comparison of participants between groups; (e) sample size; (f) outcome measures; (g) dosage and treatment duration; (h) incidence of adverse events (AE).

### 2.5. Quality Assessment of Included Studies

Two reviewers (Y. Jia and C. Chen) independently assessed the quality of the included studies according to the Jadad scale [18], the M scale [16] and the Cochrane risk of bias tool [19]. The Jadad scale focused on three criteria including “randomization,” “blinding,” and “dropouts” for assessing the quality of RCT. The M scale was modified from the Jadad scale with two additional criteria “baseline comparison of participants” and “adverse event report.” The Cochrane risk of bias tool was designed for more detailed assessment including criteria for (a) allocation concealment, (b) blinding of participants, (c) personnel and outcome assessment, (d) incomplete outcome data, (e) selective reporting, and (f) other biases [19].

### 2.6. Criteria for Symptomatic and ECG Improvements

Effective symptomatic improvements should achieve at least 50% (basic) or 80% (significant) reduction in frequency of feeling angina chest pain. Effective ECG improvements should achieve at least 0.05 mV lowering at ST segment in ECG (basic) or nearly normal (significant) ECG during an exercise test as suggested in the ACC/AHA guideline [3].

### 2.7. Meta-Analysis

Effect sizes were presented by odds ratios (OR) [20] and 95% confidence intervals (CI) [21]. Overall meta-analysis and subgroup analysis employed the random-effects model because the homogeneity of the studies could not be guaranteed. Heterogeneity among studies was assessed by chi-squared (*χ* ^2^) and *I*-squared (*I* ^2^) [22].

### 2.8. Subgroup and Sensitivity Analysis

Subgroup analysis was conducted to evaluate the overall effects in possible subgroups which were grouped according to the characteristics of the studies, including year of publication, number of authors, follow-up period, sample sizes, study periods, quality of the studies (M score), and improvements in SYMPTOMS and ECG. Mann-Whitney-Wilcoxon test was used to analyze the difference between subgroups. Sensitivity analyses were performed after excluding some studies to assess the influence of such studies on the overall result of this meta-analysis.

### 2.9. Metaregression and Risk of Bias across Studies

Funnel plots [23], Begg's rank correlation test [24], and Egger's linear regression test [25] were employed to assess the publication bias. Metaregression [26] was conducted to find the possible relationship between the efficacy and the factors such as sample sizes, M score, years of publication, and quality of RCTs.

### 2.10. Adverse Events

AEs of RCTs including nonreported AEs, types and frequency of AEs reported were surveyed for incidence of AEs during XXK and ISDN treatments.

### 2.11. Statistical Analysis

Meta-analysis and forest plot generation were performed with Review Manager 5 [27]. Metaregression and funnel plot generation was conducted with Stata software version 11 (StataCorp LP, USA). Statistical software R [28] was used to perform conventional statistical analysis, including Spearman correlation and Mann-Whitney-Wilcoxon unpaired two-sample test. *P* values lower than 0.05 were considered statistically significant.

## 3. Results

### 3.1. Study Selection

**Figure 1:**
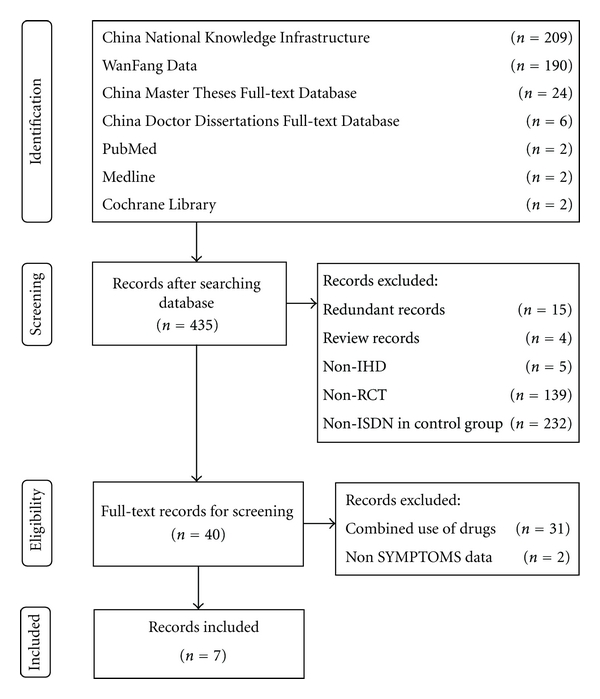
Process of searching and screening studies.

The process of study selection was depicted in Figure 1. The database search found 435 records, including 209 records from CNKI, 190 records from WanFang Data, 24 records from CMTD, 6 records from CDMD, 2 records from PubMed, 2 records from Medline, and 2 records from the Cochrane Library. According to the prespecified selection criteria described in the section Methods, only seven studies [29–35] were found eligible for further quality assessment and meta-analysis.

### 3.2. Study Characteristics

Table 1 lists the main characteristics of the included studies. All of the studies were published in Chinese language between 1993 and 2007 with a total of 550 participants. The mean sample size was 78.6 (median = 60.0; 95% CI: 52.2, 105.0). The follow-up periods were all 28 days. The daily dosage of XXK was 6 pills in all studies. The daily dosages of ISDN were 30 mg in five studies, 40 mg and 60 mg in the other two studies. All seven included studies reported SYMPTOMS changes as outcome measures while five studies [29, 31–33, 35] also reported ECG changes.

### 3.3. Quality Assessment of Included Studies

Table 1 lists the results of quality assessment according to the Jadad and M scale. All included studies were scored 2 according to the Jadad scale (between 0 and 5 points), in consistency with the average quality of Chinese RCTs [36].

According to M scale, four RCTs scored three, two RCTs scored two, and one RCT scored one. Four included studies [29, 31, 32, 35] reported baseline comparison of participants in experiment and control group. Three included studies [30, 32, 35] did not report AEs. One included study [29] reported types of AEs. Three included studies [31, 34, 35] reported types and number of AEs.

Cochrane risk of bias tool also gave similar ratings, that is, (a) unclear bias in random sequence generation; (b) high bias in allocation concealment; (c) high bias in blinding of participants; (d) personnel and outcome assessment; (e) low bias in incomplete outcome data; (f) low bias in selective reporting; (g) unclear bias in other bias.

### 3.4. Individual Studies and Their Synthesis

**Figure 2:**
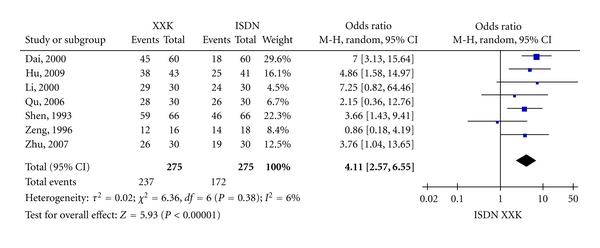
Forest plot of outcome measure SYMPTOMS.

**Figure 3:**
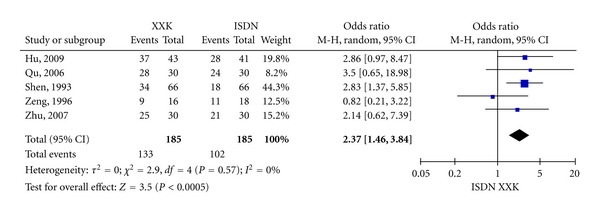
Forest plot of outcome measure ECG.

As shown in Figure 2, the overall OR of SYMPTOMS was 4.11 (95% CI = 2.57–6.55, *Z* = 5.93, *P* < 0.00001) with a heterogeneity (*τ* = 0.02, *I* ^2^ = 6%, *P* = 0.38) among the seven studies with SYMPTOMS outcome.  Figure 3 shows that the overall OR of ECG was 2.37 (95% CI = 1.46–3.84, *Z* = 3.50, *P* = 0.0005) with a heterogeneity (*τ* = 0, *I* ^2^ = 0%, *P* = 0.57) among the five studies with ECG outcome. Both the ORs of SYMPTOMS and ECG indicated that XXK is more effective than ISDN in treating angina pectoris. The overall OR of SYMPTOMS was bigger than that of ECG although the correlation in ORs between SYMPTOMS and ECG was not statistically significant (*P* = 0.6833).

### 3.5. Subgroup and Sensitivity Analysis

Based on study characteristics including M score (≤3 or >3), years of publication (before or after 1 January 2005), number of authors (1 or >1), dates of trial (reported or non-reported), baseline comparison of participants (yes or no), sample sizes (<78 or ≥78), and reports of AEs (yes or no), subgroup analysis or sensitivity analysis were performed and their results are shown in Tables 2 and 3. Wilcoxon test for comparing the ORs between subgroups in terms of SYMPTOMS and ECG found no statistically significant differences.

 When the improvements in SYMPTOMS were raised to a significant level from the basic level, the results remain effective and statistically significant (OR = 1.83, 95% CI = 1.20–2.82, *Z* = 2.77, *P* = 0.006).The overall ECG OR decreased from 2.37 to 1.60 (95% CI = 0.98–2.60, *Z* = 1.88, *P* = 0.06) with a heterogeneity (*τ* = 2.38, *I* ^2^ = 0%, *P* = 0.67). The results indicated there was not heterogeneity. Likewise, results of Spearman correlation analysis indicated there was not statistical significance (*P* = 0.95) between them.

### 3.6. Metaregression

Table 4 shows the results of metaregression between log OR and study characteristics. As the results of metaregression were not statistically significant, there seems to be no obvious correspondence of the efficacy to any study characteristic.

### 3.7. Risk of Bias across Studies

**Figure 4:**
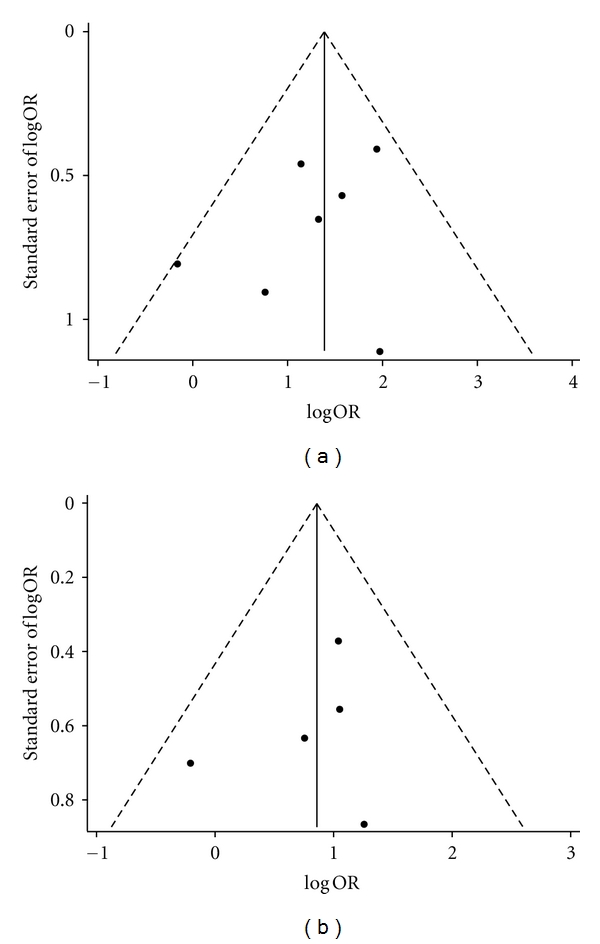
Funnel plots of (a) the included studies with SYMPTOMS data and (b) the included studies with ECG data.

Publication biases were assessed by funnel plots (Figure 4), which found no obvious asymmetry for SYMPTOMS and ECG. The Begg's test (SYMPTOMS: *Z* = −0.75, *P* = 0.453; ECG: *Z* = −0.98, *P* = 0.327) and the Egger's test (SYMPTOMS: *t* = −1.15, *P* = 0.301; ECG: *t* = −0.70, *P* = 0.536) also found no statistically significant publication bias.

### 3.8. Adverse Events

Three studies [30, 32, 35] of seven included studies did not report AEs. The AEs among 155 participants in the studies with AE reports were mainly headache and dizziness. A study [29] reported that the AE incidence of ISDN was 22.22% (4/18) and the AE incidence of XXK was 12.50% (2/16). Another study [34] reported that of the AE incidence of ISDN was 13.33% (4/30) and the AE incidence of XXK was 0% (0/30). The third study [33] reported that the AE incidence of ISDN was 28.79% (19/66) and the AE incidence of XXK was 0% (0/66). The last study [31] reported no AE for all participants. The overall AE incidence of XXK at 1.30% (2/155) was lower than that of ISDN at 17.42% (27/155).

## 4. Discussion

This study provides a PRISMA-compliant systematic review and meta-analysis based on the RCTs of XXK and ISDN to evaluate the efficacy in treating angina pectoris. Seven RCTs with 550 participants were found eligible and thus included. Overall ORs of SYMPTOMS and ECG were 4.11 and 2.37, respectively and were statistically significant (*P* < 0.05). Subgroup analysis, sensitivity analysis, and metaregression on various parameters showed this positive evidence to be robust. This meta-analysis found that XXK seems to be more effective than ISDN in treating angina pectoris.

 This systematic review is comprehensive, reliable, and PRISMA-compliant. In this meta-analysis, (a) a comprehensive search for RCTs published between 1989 and 2011 were performed; (b) only the studies with XXK and ISDN as single-drug interventions were included; (c) subgroup analysis, sensitivity analysis, and metaregression were performed according to the basic characteristics of included studies; (d) publication bias was assessed not only by funnel plots but also by the Begg's test and Egger's test.

This study indicated XXK seems to be more effective than ISDN in treating angina pectoris. The active components of XXK are steroidal saponin glycosides, among which Saponin DP 1–8 is a major component [37]. XXK were able to (a) maintain the activity of Ca^2+^-ATP enzyme and Na^+^-K^+^-ATP enzyme through free radicals removal; (b) protect myocardial cells from ischemia and maintain the integrity of myocardial cell membrane; (c) protect myocardial cells through dilated coronary arteries, reduced coronary resistance, increased coronary blood flow, improved blood and oxygen supply to myocardium; (d) improve myocardial ischemia symptoms through reducing myocardial oxygen consumption and enhancing tolerance of myocardium to hypoxia [38, 39].

 A major limitation of this systematic review, like other systematic reviews on Chinese RCTs [16], is the quality of included studies. Only 6.8% RCTs on 20 common diseases published from January 1994 to June 2005 are authentic RCTs conducted in China National Knowledge Infrastructure electronic database [40]. Most of the included studies scored only 2 at the Jadad scale, which is a scale ranged between 0 and 5. Neither allocation concealment nor blinding was mentioned in the included studies. Four out of seven included studies scored only 3 at the M scale (ranged between 1 and 7). Although we found no statistically significant difference in the effect sizes between different grades of study quality, further updates of this systematic review should be required to confirm the efficacy of XXK over ISDN.

 All studies included in this meta-analysis did not report full outcome measure data. They only reported the results based on the number of participants with different responses to the treatments. Moreover, the follow-up period of all studies was only 28 days; thus, tolerance of ISDN over time would not be estimated. Tolerance of ISDN was not adequately considered in past meta-analyses of (even) western medicine RCTs.

 This systematic review included the studies comparing XXK and ISDN and excluded the studies with combined drugs. The dosages of XXK were in accordance with recommended dosages (two pills each time, three times a day) of the manufacturer. Daily dosages of ISDN ranged from 30 mg to 60 mg. The guideline of ACC/AHA 2002 suggested the oral dosage range of ISDN to be 5–80 mg each time twice or three times a day. A previous study [41] found that high dosages of ISDN were not better than low dosages of ISDN in treating angina pectoris. The interpretation of the trial results in this systematic review would not be complicated by the combined use of DSP or ISDN with other drugs.

 Incidence of AEs due to XXK (1.30%) was lower than those due to ISDN (17.42%). All seven included studies did not report AEs in a standard format. Three studies did not report AEs at all. Thus, the safety of XXK could not be thoroughly evaluated although it is well appreciated in the research community that RCTs should report AEs properly [42].

## 5. Conclusion

The meta-analysis of eligible but limited RCTs demonstrates that XXK seems to be more effective than ISDN in treating angina pectoris. Further RCTs of high quality are warranted to be conducted for update of the results of this meta-analysis.

## Figures and Tables

**Table 1:** Summary of the included studies evaluating the efficacy of XXK in treating angina pectoris.

Study	No. of authors	Trail date	Sample size	Follow-up period	Comparable	AEs	Outcome measures	XXK daily dosage	ISDN daily dosage	Jadad score	M score
Zeng et al. [29]	6	0	34	28 days	1	1	SYM, ECG	6 pills	40 mg	2	4
Dai [30]	1	1	120	28 days	0	0	SYM	6 pills	30 mg	2	2
Hu [31]	1	0	84	28 days	1	1	SYM, ECG	6 pills	30 mg	2	4
Li and Zhang [34]	2	1	60	28 days	0	1	SYM	6 pills	60 mg	2	3
Qu [32]	1	1	60	28 days	1	0	SYM, ECG	6 pills	30 mg	2	3
Shen [33]	1	1	132	28 days	0	1	SYM, ECG	6 pills	30 mg	2	3
Zhu et al. [35]	5	1	60	28 days	1	0	SYM, ECG	6 pills	30 mg	2	3

XXK is di'ao xinxuekang capsule; ISDN is isosorbide dinitrate; SYM is SYMPTOMS; ECG is electrocardiogram; AEs is adverse events; Numbers 1 and 0 in “Trial date” mean that study reported or did not report the trial date, respectively; Numbers 1 and 0 in “Comparable” mean that the study reported the comparability between the experiment and control groups (1) or the study did not report that (0).

**Table 2:** Subgroups and sensitivity analysis based on ORs of SYMPTOMS outcomes.

	Group	No. of studies	No. of participants	OR	Wilcoxon test	95% CI	*Z*	*P* (effect)	*I* ^2^	*χ* ^2^	*P* (het)
M score	≤3	5	432	4.76	*W* = 7	2.85, 7.95	5.96	<0.00001	0%	2.21	0.70
	>3	2	118	2.24	*P* = 0.4386	0.41, 12.17	0.94	0.35	67%	3.06	0.08
Sample size	<78	4	214	2.49	*W* = 4	1.09, 5.68	2.17	0.03	3%	3.09	0.38
	≥78	3	336	5.22	*P* = 0.4795	3.05, 8.93	6.03	<0.00001	0%	1.07	0.59
No. of authors	1	4	396	4.85	*W* = 6	2.90, 8.11	6.01	<0.00001	0%	1.94	0.58
	>1	3	154	2.61	*P* = 1	0.82, 8.36	1.62	0.11	35%	3.06	0.22
Publication year	<2005	4	346	3.84	*W* = 7	1.65, 8.93	3.12	0.002	47%	5.71	0.13
	≥2005	3	204	3.82	*P* = 0.7237	1.78, 8.21	3.44	0.0006	0%	0.58	0.75
Trial date	Reported	5	432	4.76	*W* = 7	2.85, 7.95	5.96	<0.000 01	0%	2.21	0.70
	Not reported	2	118	2.24	*P* = 0.4386	0.41, 12.17	0.94	0.35	67%	3.06	0.08
Comparable	Reported	4	238	2.83	*W* = 2	1.36, 5.89	2.78	0.005	10%	3.35	0.34
	Not reported	3	312	5.45	*P* = 0.1573	3.02, 9.83	5.64	<0.00001	0%	1.12	0.57
AEs	Reported	4	310	3.30	*W* = 6	1.58, 6.88	3.18	0.0001	21%	3.78	0.29
	Not reported	3	240	5.17	*P* = 1	2.74, 9.78	5.06	<0.00001	0%	1.71	0.43
Improvement	>50%	7	550	4.11	*W* = 7	2.57, 6.55	5.93	<0.00001	6%	6.36	0.38
	>80%	5	370	1.83	*P* = 0.2506	1.20, 2.82	2.77	0.006	0%	1.10	0.89

CI is confidence interval. Mann-Whitney-Wilcoxon test was performed on ORs of SYMPTOMS. *Z* and *P* (effect) evaluated the statistics of overall effect; *I* ^2^, *χ* ^2^, and *P* (het) were computed to assess heterogeneity.

**Table 3:** Subgroups and sensitivity analysis based on ORs of ECG outcomes.

	Group	No. of studies	No. of participants	OR	Wilcoxon test	95% CI	*Z*	*P* (effect)	*I* ^2^	*χ* ^2^	*P* (het)
M score	≤3	3	252	2.73	*W* = 4	1.52, 4.91	3.35	0.0008	0%	0.24	0.89
	>3	2	118	1.65	*P* = 0.5637	0.49, 5.57	0.8	0.42	49%	1.98	0.16
Sample size	<78	3	154	1.71	*W* = 2	0.77, 3.84	1.31	0.19	0%	1.93	0.38
	≥78	2	216	2.84	*P* = 0.5637	1.56, 5.20	3.39	0.0007	0%	0	0.99
No. of authors	1	3	276	2.91	*W* = 6	1.65, 5.14	3.69	0.000 2	0%	0.05	0.97
	>1	2	94	1.39	*P* = 0.0833	0.54, 3.55	0.68	0.5	4%	1.05	0.31
Publication year	<2005	2	166	1.75	*W* = 1	0.54, 5.74	0.93	0.35	60%	2.47	0.12
	≥2005	3	204	2.69	*P* = 0.2482	1.29, 5.60	2.63	0.008	0%	0.24	0.89
Trial date	Reported	2	118	1.65	*W* = 2	0.49, 5.57	0.8	0.42	49%	1.98	0.16
	Not reported	3	252	2.73	*P* = 0.5637	1.52, 4.91	3.35	0.0008	0%	0.24	0.89
Comparable	Reported	4	238	2.06	*W* = 2	1.08, 3.93	2.19	0.03	0%	2.48	0.48
	Not reported	1	132	2.83	*P* = 1	1.37, 5.85	2.81	0.005	NA	NA	NA
AEs	Reported	3	250	2.22	*W* = 2	1.14, 4.32	2.33	0.02	25%	2.66	0.26
	Not reported	2	120	2.54	*P* = 0.5637	0.94, 6.90	1.83	0.07	0%	0.21	0.65
Improvement	>50%	5	370	2.37	*W* = 14	1.46, 3.84	3.5	0.0005	0%	2.90	0.57
	>80%	5	370	1.60	*P* = 0.7540	0.98, 2.60	1.88	0.06	0%	2.38	0.67

CI is confidence interval. NA is not available; Mann-Whitney-Wilcoxon test was performed on ORs of ECG. *Z* and *P* (effect) evaluated the statistics of overall effect; *I* ^2^, *χ* ^2^, and *P* (het) were computed to assess heterogeneity.

**Table 4:** Metaregression of basic characteristics of RCTs and ORs of SYMPTOMS and ECG outcomes.

Log OR	No. of RCTs	No. of participants	Factor tested	Coefficient	*t*	*P*
			Publication year	0.0200	0.43	0.685
			No. of authors	−0.2106	−1.63	0.165
			Trail date	0.5646	1.00	0.365
SYMPTOMS	7	550	Sample size	0.0069	1.52	0.190
			Comparable	−0.6369	−1.38	0.227
			AEs	−0.4280	−0.89	0.412
			M score	−0.4829	−1.56	0.180
			Publication year	0.0097	0.26	0.809
			No. of authors	−0.1796	−1.46	0.239
			Trail date	0.4354	0.83	0.469
ECG	5	370	Sample size	0.0073	1.10	0.354
			Comparable	−0.3195	−0.64	0.565
			AEs	−0.0911	−0.16	0.886
			M score	−0.4354	−0.83	0.469

Metaregression was conducted by residual (restricted) maximum likelihood (REML) with Knapp-Hartung modification.
